# Mathematical Models of Early Hepatitis B Virus Dynamics in Humanized Mice

**DOI:** 10.1007/s11538-024-01284-2

**Published:** 2024-04-09

**Authors:** Stanca M. Ciupe, Harel Dahari, Alexander Ploss

**Affiliations:** 1 Department of Mathematics, Virginia Polytechnic Institute and State University, Blacksburg, VA USA; 2 Division of Hepatology, Department of Medicine, Loyola University, Chicago, IL USA; 3 Department of Molecular Biology, Princeton University, Princeton, NJ USA

**Keywords:** Mathematical modeling, HBV, HBsAg, Humanized mice

## Abstract

Analyzing the impact of the adaptive immune response during acute hepatitis B virus (HBV) infection is essential for understanding disease progression and control. Here we developed mathematical models of HBV infection which either lack terms for adaptive immune responses, or assume adaptive immune responses in the form of cytolytic immune killing, non-cytolytic immune cure, or non-cytolytic-mediated block of viral production. We validated the model that does not include immune responses against temporal serum hepatitis B DNA (sHBV) and temporal serum hepatitis B surface-antigen (HBsAg) experimental data from mice engrafted with human hepatocytes (HEP). Moreover, we validated the immune models against sHBV and HBsAg experimental data from mice engrafted with HEP and human immune system (HEP/HIS). As expected, the model that does not include adaptive immune responses matches the observed high sHBV and HBsAg concentrations in all HEP mice. By contrast, while all immune response models predict reduction in sHBV and HBsAg concentrations in HEP/HIS mice, the Akaike Information Criterion cannot discriminate between non-cytolytic cure (resulting in a class of cells refractory to reinfection) and antiviral block functions (of up to $99\%$ viral production 1–3 weeks following peak viral load). We can, however, reject cytolytic killing, as it can only match the sHBV and HBsAg data when we predict unrealistic levels of hepatocyte loss.

**Supplementary Information:**

The online version contains supplementary material available at 10.1007/s11538-024-01284-2.

## Introduction

Our current understanding of key immunological interactions and molecular dynamics responsible for the early stages of hepatitis B virus (HBV) infection is largely based on mathematical modeling validated against virus titer data in the serum of infected patients and chimpanzees Ciupe et al. (2007b, 2014, 2007a); Murray et al. (2005); Forde et al. (2016). These models have provided information about early HBV dynamics and the potential role of immune system in viral clearance or establishment of chronic disease. They showed that resolution of acute infections, where high levels of up to $10^{10}$ HBV DNA copies per ml and potentially up to $95\%$ infections in adults are spontaneously cleared within 3-6 months, requires a broad and vigorous adaptive immune response. However, the exact interplay and contribution of humoral and cellular immune responses remain unknown Ciupe (2018); Ferrari et al. (1990). The humoral immune response yields virus specific antibodies capable of neutralizing infectious HBV and can thereby protect hepatocytes from new infections Glebe et al. (2009); Rath and Devey (1988). Anti-HBV antibodies have been shown to play a role only in the final resolution on the infection Ciupe et al. (2014). By contrast, CD8 T cell-mediated immune responses have been shown to remove infected cells through cytolytic killing and permanently inactivating HBV in cells through non-cytolytic mechanisms (such as the production of cytokines) McClary et al. (2000); Guidotti et al. (1996); Wieland et al. (2004). Such cellular adaptive immune responses have been shown to play a role in both controlling overall viremia and contributing to HBV clearance Ciupe et al. (2007b); Murray et al. (2005); Ciupe et al. (2007a). Notably, modeling work postulates that virus resolution through non-cytolytic mechanisms requires that cured cells remain refractory to reinfection by the (still) abundant virus Ciupe et al. (2007a). Lastly, mathematical models have shown that the size of inoculum dose has an effect on both the timing of the CD8 T cell expansion and the quality of its response, especially its non-cytolytic function Ciupe et al. (2021), hence explaining the observed relationship between inoculum size and infection outcome in HBV-infected chimpanzees Asabe et al. (2009).

Despite these advances, the lack of immunological and molecular data in the early acute phase of the infection has hampered our understanding of the mechanistic interactions that determine successful viral expansion, infection outcome, and (later on) treatment response. The intricate interplay between the virus and the immune system requires quantification of additional data on viral markers such as serum hepatitis B surface-antigen (HBsAg), serum hepatitis B e-antigen (HBeAg), serum HBV DNA (sHBV), various HBV nucleic acid replication intermediates - covalently closed circular DNA(cccDNA), intracellular HBV DNA, pre-genomic RNA (pgRNA) - as well as phenotyping and functional testing of antiviral T cell responses.

The recent establishment of humanized mice, i.e. mice expressing human genes and/or engrafted with human tissue, has provided access to early measurements of virological and molecular markers. Mice are either singly engrafted with human hepatocytes (HEP) Hogan et al. (2023); Gutti et al. (2014); Dusséaux et al. (2017) or dually co-engrafted with human hepatocytes and components of a human immune system (HEP/HIS) Hogan et al. (2023); Billerbeck et al. (2016); Dusséaux et al. (2017) before being challenged with HBV. The humanized human liver provides the necessary environment for HBV, a uniquely human-, hepatotropic virus. Co-engraftment of components of a human immune system enables tracking of human immunity in responses to the viral infection in the liver. In this study, we analyzed data from two groups of either HEP or HEP/HIS mice challenged with HBV for which longitudinal sHBV and longitudinal HBsAg titers were collected biweekly for up to six weeks following infection Hogan et al. (2023). HBsAg is believed to serve as both decoy against humoral immune responses Ciupe et al. (2014) and to induce T-cell exhaustion Kim et al. (2020); Fang et al. (2015); Bertoletti and Gehring (2006). Mice in the HEP/HIS group had lower sHBV and HBsAg levels than HEP mice, suggesting that the grafted human immune system mounts an antiviral response, which can, at least partially, control HBV infection Hogan et al. (2023). Several mechanisms may explain the observed differences: either (partial) loss/death of productively HBV-infected cells i.e. cytolytic killing or non-cytolytic suppression of HBV replication. In case of cytolytic elimination of HBV infection, immune inflicted liver damage would trigger proliferation of naÃ¯ve hepatocytes, which in turn could be targets for *de novo* infection by circulating HBV, thereby establishing a dynamic equilibrium. To explore the mechanisms or combined mechanisms responsible for the observed differences in the sHBV and HBsAg dynamics in the HEP and HEP/HIS groups, we developed mathematical models of HBV infection and validated them against sHBV and HBsAg titer data.

## Mathematical Model

**Fig. 1:**
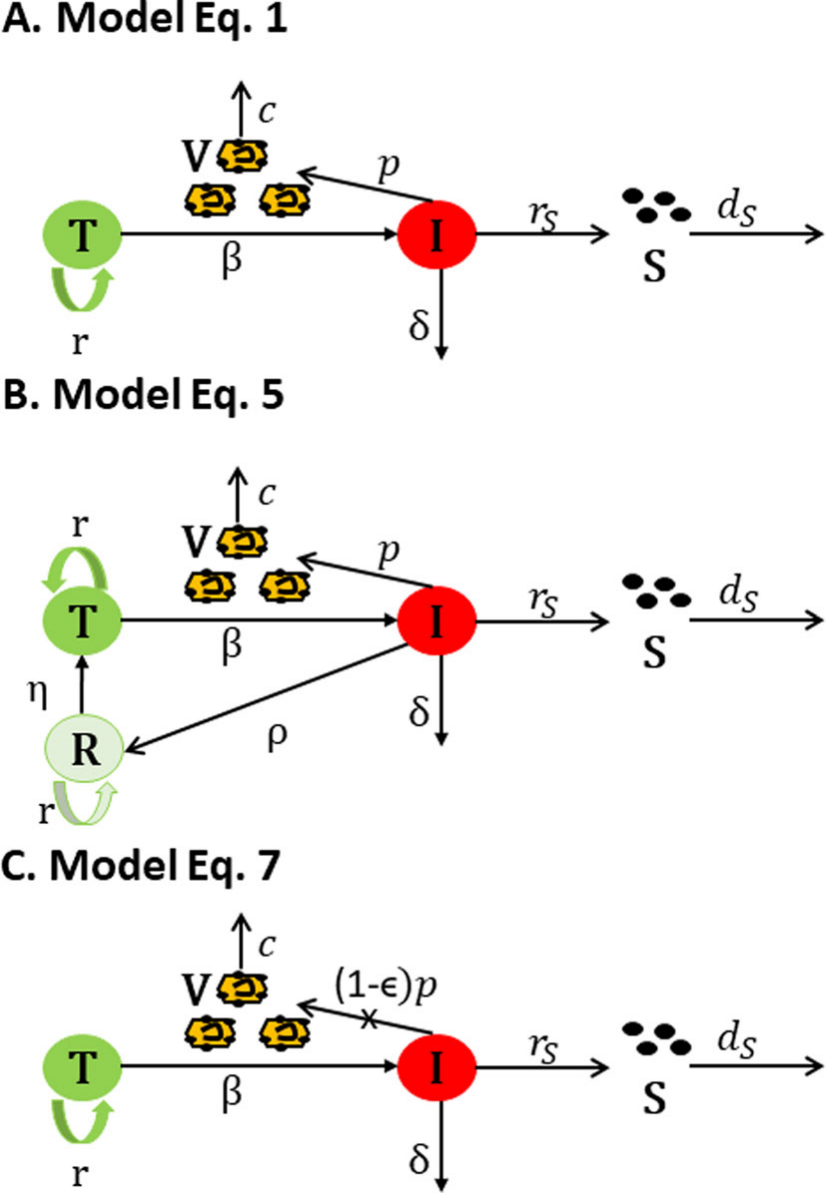
Model description. **A**: Model of HBV infection with cytotoxic immune responses given by Eq. 1; **B**. Model of HBV infection with non-cytotoxic immune responses given by Eq. 6; and **C**: Model of HBV infection with antiviral effects given by Eq. 8

We utilized a within-host model of HBV infection that considers the interaction between target liver cells, *T*, infected liver cells, *I*, HBV, *V*, and HBsAg, *S*, as follows Ciupe et al. (2007b, 2007a); Kadelka et al. (2021). Target cells, *T*, interact with the virus, *V*, at rate $\beta$ to become infected cells, *I*. Infected cells die at per capita rate $\delta$, and produce virus at rate *p*. Virus is cleared at rate *c*. Uninfected cells are maintained through homeostasis. We model this using a logistic term with maximum per capita growth rate *r* and carrying capacity *K*. Lastly, HBsAg, *S* are produced at rate $r_S$ (proportional to the infected cell density) and decay at per capita rate $d_S$. The diagram describing these interactions is shown in Fig. 1A and the interactions are given by the following system,

1 $$\begin{aligned} \begin{aligned} \frac{dT}{dt}&=rT(1-\frac{T+I}{K}) -\beta T V,\\ \frac{dI}{dt}&= \beta T V -\delta I,\\ \frac{dV}{dt}&= p I - cV, \\ \frac{dS}{dt}&=r_S I- d_S S, \end{aligned} \end{aligned}$$

with initial conditions $T(0)=K$, $I(0)=0$, $V(0)=V_0$ and $S_0=S_0$. We use model Eq. 1 to understand the mechanistic interactions responsible for the differences between HBV infections in HEP and HEP/HIS mice.

## Data Fitting

### Empirical Data

Humanized mice were generated by engrafting human hepatocytes (HEP mice) or hepatocytes and human immune cells (HEP/HIS mice) into immunodeficient xenorecipient strains (for details please see Hogan et al. (2023)). Human hepatic and/or hematopoietic engraftment was quantified prior to infection with HBV. Groups of HEP mice (n = 7) and HIS/HEP mice (n = 12) were infected intravenously with cell-culture produced HBV (1x10E6 GE/mouse, genotype D, strain ayw). Blood was sampled prior to and at weeks 2, 4 and 6 post infection. At week 6, all mice were culled to harvest blood, spleens and livers for analysis. Several virological markers (HBeAg, HBsAg, sHBV) as well as human albumin was quantified in the serum by ELISA and qPCR. HBV DNA, pgRNA, and cccDNA were quantified in liver tissue by (RT)qPCR. Frequencies and phenotypes of human lymphocytes were measured in the blood, spleens and livers by flowcytometry Hogan et al. (2023). For the modeling purposes we only used sHBV and HBsAg titers, for which we have temporal data above limit of detection.

### Parameter Estimation

We assume that $K=6.8\times 10^5$ hepatocytes/ml are susceptible to HBV infection (20-times lower than in humans Ciupe et al. (2007a)), their per capita division rate is $r=1$ per day Ciupe et al. (2007b), virus is cleared at rate $c=4.4$ per day Murray et al. (2006), and HBsAg decays at rate $d_S=0.01$ per day Kadelka et al. (2021). The initial conditions are $T(0)=K$ per ml, $I(0)=0$ per ml, $V(0)=100$ per ml (the virus limit of detection) and $S(0)=10^{-6}$ per ml (below the HBsAg limit of detection). For the mice in the HEP group (i.e. without human immune response) we estimate parameters ${par}_{HEP}=\{\beta , r_S, p\}$ by fitting theoretical curves for *V*(*t*) and *S*(*t*) as given by Eq. 1 with $\delta =0$ to sHBV and HBsAg empirical data. For the mice in the HEP/HIS group, we estimate parameters ${par}_{HEP/HIS}=\{\beta , r_S, \delta , p\}$ by fitting theoretical curves for *V*(*t*) and *S*(*t*) as given by Eq. 1 to sHBV and HBsAg empirical data. For a description of model parameters see Table 1.

**Table 1:** Parameters description

Parameter	Description	Value	References
*r*	Hepatocyte division rate	1 d^-1^	Ciupe et al. (2007b)
*K*	Hepatocyte carrying capacity	$6.8\times 10^5$ hep/ml	Ciupe et al. (2007a)
$\beta$	Infectivity rate	estimated	–
$\delta$	Killing rate	estimated	–
*p*	Viral production rate	estimated	–
*c*	Virus clearance rate	4.4 d^-1^	Murray et al. (2005)
$\rho$	Recovery rate	0.05 d^-1^	–
$\eta$	Waning rate	0.001 d^-1^	Ciupe et al. (2007a)
$r_S$	HBsAg production rate	estimated	–
$d_S$	HBsAg decay rate	0.01 d^-1^	Kadelka et al. (2021)

Initial Condition	Description	Value	Reference
*T*(0)	Healthy hepatocytes	*K* hep/ml	–
*I*(0)	Infected hepatocytes	0 hep/ml	–
*R*(0)	Recovered hepatocytes	0 hep/ml	- -
*V*(0)	Initial virus	100 virion/ml	–
*S*(0)	Initial HBsAg	$10^{-6}$ HBsAg/ml	–

**Fig. 2:**
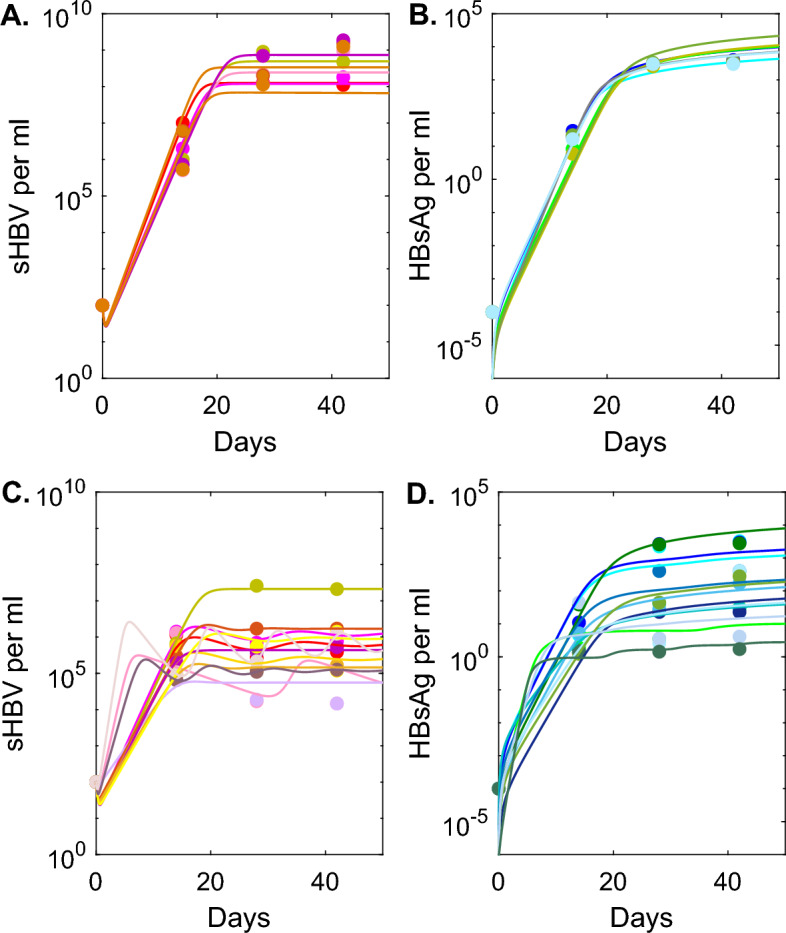
**A**, **B**: *V*(*t*) and *S*(*t*) as given by model Eq. 1 with $\delta =0$ versus HEP data; **C**, **D**: *V*(*t*) and *S*(*t*) as given by model Eq. 1 with $\delta \ne 0$ versus HEP/HIS data. Model parameters are given in Table 3 (Color figure online)

### Fitting Method

We validate model Eq. 1 against individual mouse sHBV and HBsAg data in either the HEP or HEP/HIS mouse groups, as follows. We estimate unknown parameters ${par}_{HEP}$ by fitting both *V*(*t*) and *S*(*t*) given by Eq. 1 with $\delta =0$ to the individual HEP mouse sHBV and HBsAg data, simultaneously. We estimate unknown parameters ${\textbf {par}}_{HEP/HIS}$ by fitting both *V*(*t*) and *S*(*t*) given by Eq. 1 with $\delta \ne 0$ to the individual HEP/HIS mouse sHBV and HBsAg data, simultaneously. We define the objective functional

2 $$\begin{aligned} \begin{aligned} J(\mathbf {par_n})=\left( \sum _{j=1}^4(\log _{10} V^i(j)-\log _{10} V^i_{d}(j)\right) ^{1/2}\\ +\left( \sum _{j=1}^{4}(\log _{10} S^i(j)- \log _{10} S^i_{d}(j))^2\right) ^{1/2}, \end{aligned} \end{aligned}$$

for each animal *i*. Here *V*(*j*) is the virus curve given by model Eq. 1 at day *j* post infection, $V_{d}(j)$ is the sHBV data at day *j* post infection, *S*(*j*) is the HBsAg curve given by model Eq. 1 at day *j* post infection, $S_{d}(j)$ is the HBsAg data at day *j* post infection, $j=\{0, 14, 28, 42\}$ days, and $n=\{\text {HEP}, \text {HEP/HIS}\}$. We minimize $J(\mathbf {par_n})$ over the parameter space using the *fminsearch* function in MATLAB. The results for the HEP mice are shown in Fig. 2A, B and the best parameter fits are given in Table 2. Similarly, the results for the HEP/HIS mice are shown in Fig. 2C, D and the best parameter fits are given in Table 3.

Additionally, to address heterogeneity in the HEP/His data, we estimate population level mean and standard deviation for parameters $\textbf{p}=\{\beta , r_S, \delta , p\}$ for model Eq. 1 using a non-linear mixed effects modelling approach that utilizes Stochastic Approximation Estimation-Maximization (SAEM) algorithm in Monolix Monolix version 2019r2 (2019) (see Fig. S1 and table S1).

**Table 2:** Parameter estimates found by fitting model Eq. 1 with $\delta =0$ to sHBV and HBsAg data for the HEP group

HEP group Eq. 1$\delta =0$				
mouse id	$\beta \times {10^{-9}}$	$r_S\times 10^{-4}$	*p*	RSS
	ml/(vir.$\times$ d)	1/(inf. cell$\times$ d)	1/(inf. cell$\times$ d)	
229	9.24	2.24	813	0.3
14045	8.83	5.23	768	0.73
14055	4.0	5.74	1580	1.26
14056	2.0	6.42	3190	0.93
14058	3.6	3.65	2190	1.04
14064	1.3	13	4790	1.7
14068	14.8	3.71	448	1.73
mean	6.27	5.71	1970	
median	4.04	5.23	1580

**Table 3:** Parameter estimates found by fitting model Eq. 1 with $\delta \ne 0$ to sHBV and HBsAg data for the HEP/HIS group

HEP/HIS group Eq. 1, $\delta \ne 0$					
mouse id	$\beta \times {10^{-7}}$	$r_S\times 10^{-4}$	$\delta$	*p*	RSS
	ml/(vir.$\times$ d)	1/(inf. cell$\times$ d)	1/d	1/(inf. cell$\times$ d)	
	ml/(vir.$\times$ d)	1/(inf. cell$\times$ d)	1/d	1/(inf. cell$\times$ d)	
3205	7.27	1.33	0.155	9.8	1.78
3206	3.77	2.09	0.172	19.8	1.26
3346	62.1	0.01	0.126	2.54	1.85
14051	0.43	4.24	0	138	0.67
14062	26.8	1.09	7.46	21.6	1.54
8061	2.00	0.14	0.775	54.7	0.46
8044	17.1	0.24	0.157	3.85	1.71
669	19.7	0.06	0	2.82	0.49
621	22.8	0.09	0.599	3.96	1.49
623	4.23	0.23	0.203	14.6	0.62
661	46.2	0.03	0.501	3.25	1.2
612	10.0	0.006	0.473	27.8	0.33
mean	18.5	0.79	0.18	25.3	
median	13.6	0.18	0.18	12.2	

## Results

### Analytical Results

Model Eq. 1 has three equillibria, the no-liver equilibrium

$$\begin{aligned} E_0=(0, 0, 0, 0), \end{aligned}$$

which is not biologically realistic; the disease free equilibrium

$$\begin{aligned} E_1=(K, 0,0,0), \end{aligned}$$

and the endemic equilibrium

$$\begin{aligned} E_2=\left( \frac{c\delta }{\beta p}, \frac{r-\frac{c\delta r}{\beta p K}}{\frac{r}{K}+\beta \frac{p}{c}}, \frac{p(r-\frac{c\delta r}{\beta p K})}{c(\frac{r}{K}+\beta \frac{p}{c})}, \frac{r_S(r-\frac{c\delta r}{\beta p K})}{d_S(\frac{r}{K}+\beta \frac{p}{c})}\right) , \end{aligned}$$

which exists iff and only if

$$\begin{aligned} R_0=K\frac{\beta p}{c\delta }>1. \end{aligned}$$

#### Proposition 1

The disease free equilibrium $E_1$ is locally asymptotically stable iff $R_0<1$ and unstable otherwise.

#### Proof

The Jacobian of Eq. 1 at equilibrium $\bar{E}=(\bar{T}, \bar{I}, \bar{V}, \bar{S})$ is

$$\begin{aligned} J( \bar{E})=\left( \begin{matrix} r-\frac{r}{K}\bar{I}-2\frac{r}{K}\bar{T}-\beta \bar{V} &{} -\frac{r}{K}\bar{T} &{} -\beta \bar{T}&{} 0\\ \beta \bar{V} &{} -\delta &{} \beta \bar{T} &{} 0\\ 0 &{} p &{} -c&{}0\\ 0&{} r_S &{}0 &{}-d_S \ \end{matrix}\right) . \end{aligned}$$

**Table 4:** Parameter estimates found by fitting model Eq. 6 with $\rho =0.05$ per day to sHBV and HBsAg data for the HEP/HIS group

HEP/HIS group					
mouse id	$\beta \times {10^{-7}}$	$r_S\times 10^{-4}$	$\delta$	*p*	RSS
	ml/(vir.$\times$ d)	1/(inf. cell$\times$ d)	1/d	1/(inf. cell$\times$ d)	
3205	2.27	2.65	0.07	29.3	1.76
3206	3.27	1.96	0	20.3	1.25
3346	15	0.02	0.11	6.02	1.51
14051	0.23	6.72	0	290	0.76
14062	7.18	0.12	0.26	10.8	1.17
8061	3.04	0.04	0	21.5	0.68
8044	4.86	0.46	0.09	13.4	1.57
669	8.64	0.12	0	7.1	0.82
621	21.5	0.04	0	2.5	1.74
623	4.11	0.18	0	12.5	0.90
661	60	0.01	0	2.4	1.76
612	10.0	0.003	0.02	16.9	1.62
mean	26.7	1.0	0.05	36.2	
median	7.9	0.12	0.00002	13.2	

**Fig. 3:**
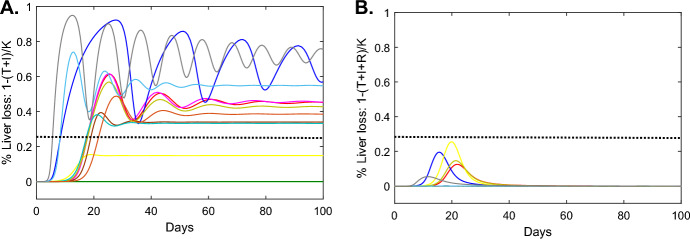
**A**: Total liver loss $1-(T+I)/K$ predicted by model Eq. 1 with $\delta \ne 0$ for the HEP/HIS group; **B**: Total liver loss $1-(T+I+R)/K$ predicted by model Eq. 6 for the HEP/HIS group. Model parameters are given in Tables 3 and 4. Dashed black line accounts for an arbitrary chosen liver loss level of $30\%$, which we assume to be non-life threatening (Color figure online)

**Fig. 4:**
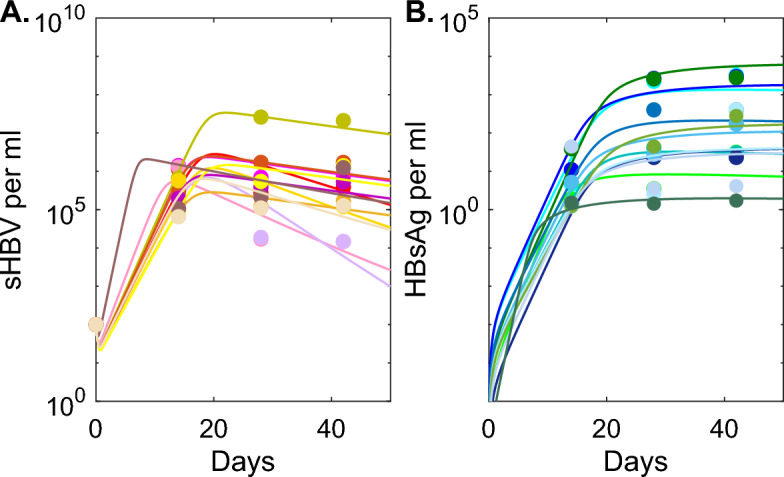
**A**: *V*(*t*) as given by model Eq. 6 versus HEP/HIS sHBV data; **B** *S*(*t*) as given by model Eq. 6 versus HEP/HIS HBsAg data. Model parameters are given in Table 4 and $\rho =0.05$ per day (Color figure online)

The Jacobian evaluated at $E_1$ becomes

$$\begin{aligned} J( E_1)=\left( \begin{matrix} -r&{} -r &{} -\beta K &{} 0\\ 0 &{} -\delta &{} \beta K &{} 0\\ 0 &{} p &{} -c&{}0\\ 0&{} r_S &{}0 &{}-d_S \ \end{matrix}\right) , \end{aligned}$$

with eigenvalues $\lambda _1=-d_S<0$, $\lambda _2=-r$, and

$$\begin{aligned} \lambda _{3,4}=\frac{-(c+\delta )\pm \sqrt{(c+\delta )^2-4(c\delta -\beta pK)}}{2}<0 \end{aligned}$$

iff $R_0=K\frac{\beta p}{c\delta }<1$. Hence, equilibrium $E_1$ is locally asymptotically stable if $R_0<1$ and unstable otherwise. $\square$

#### Proposition 2

The endemic equilibrium $E_2$ is locally asymptotically stable if $R_0>1$ and $(\delta ^2 + (3c + r)\delta + c^2)(\delta R_0+r)R_0 + c (r^2-R_0^3 \delta ^2)>0$ and unstable otherwise.

#### Proof

The Jacobian of Eq. 1 evaluated at $E_2$

$$\begin{aligned} J( E_2)=\left( \begin{matrix} -\frac{r}{R_0}&{} -\frac{r}{R_0} &{} -\frac{c\delta }{p} &{} 0\\ \frac{r\delta (R_0-1)}{r\delta +R_0} &{} -\delta &{} \frac{c\delta }{p} &{} 0\\ 0 &{} p &{} -c&{}0\\ 0&{} r_S &{}0 &{}-d_S \ \end{matrix}\right) , \end{aligned}$$

has eigenvalues $\lambda _1=-d_S<0$ and $\lambda _{2,3,4}$ which solve the equation

$$\begin{aligned} \lambda ^3+A_1\lambda ^2+A_2\lambda +A_3=0, \end{aligned}$$

with

3 $$\begin{aligned} \begin{aligned} A_1&=c+\delta +\frac{r}{R_0},\\ A_2&=\frac{(\delta (c + r + \delta ) R_0 + cr)r}{R_0 (R_0\delta + r)},\\ A_3&=\frac{(R_0 - 1)c\delta r}{R_0}. \end{aligned} \end{aligned}$$

By the Routh Hurwitz condition, eigenvalues $\lambda _{2,3,4}$ have negative real part if $A_1>0$ (always true), $A_2>0$ (always true), $A_3>0$ (true when $R_o>1$) and $A_1 A_2-A_3>0$. It is easy to show that $A_1 A_2-A_3>0$ when $(\delta ^2 + (3c + r)\delta + c^2)(\delta R_0+r)R_0 + c (r^2-R_0^3 \delta ^2)>0$. This concludes our proof. $\square$

Hence, in the long-run virus *V*(*t*) and antigen *S*(*t*) given by system Eq. 1 will either

1. Asymptotically reach zero, symbolizing clearance of infection, if $R_0<1$;
2. Reach the endemic equilibrium $E_2$ when $R_0>1$ and $(\delta ^2 + (3c + r)\delta + c^2)(\delta R_0+r)R_0 + c (r^2-R_0^3 \delta ^2)>0$;
3. Oscillate around the chronic equilibrium $E_2$ when $R_0>1$ and $(\delta ^2 + (3c + r)\delta + c^2)(\delta R_0+r)R_0 + c (r^2-R_0^3 \delta ^2)<0$.

### Numerical Results

We found homogeneous dynamics within the HEP group for both sHBV (see Fig. 2A) and HBsAg (see Fig. 2B) curves. We predict low viral infectivity rate $\beta =6.3\times 10^{-9}$ ml/virus per day and large viral production $p=1970$ virion per day. The HBsAg expansion rate is similar among the mice, with average $r_S=5.7 \times 10^{-4}$ HBsAg being produced per infected cell per day.

**Table 5:** Parameter estimates found by fitting model Eq. 8 to sHBV and HBsAg data for the HEP/HIS group

HEP/HIS group						
mouse id	$\beta \times {10^{-7}}$	$r_S\times 10^{-4}$	$\epsilon _0$	$\tau$	*p*	RSS
	ml/(vir.$\times$ d)	1/(inf. cell$\times$ d)		d	1/(inf. cell$\times$ d)	
3205	0.43	1.75	0.99	25.6	141	0.51
3206	1.12	1.50	0.91	25.5	57	0.53
3346	8.15	0.01	0.98	14	9.1	0.56
14051	0.43	4.24	0.00	0	138	0.63
14062	1.94	0.16	0.98	15	28	0.45
8061	4.99	0.03	0.58	48	11	0.46
8044	6.63	0.15	0.91	25.5	9.2	0.9
669	12.6	0.06	0.63	5.2	7.9	0.46
621	7.75	0.07	0.79	13.9	6.2	1.25
623	1.60	0.36	0.70	10.6	35.1	0.53
661	10.0	0.006	0.99	11.6	84.6	1.38
612	10.0	0.001	0.99	11	169	1.07
mean	6.88	0.7	0.79	17.2	58.1	
median	5.81	0.1	0.91	13.9	31.6	

By contrast, both sHBV and HBsAg dynamics within the HEP/HIS mice are more heterogeneous (see Fig. 2C and D), with mice 14051 and 669 having no indication of infected cell death $\delta =0$ per day and the rest having an average infected cell killing rate $\delta =0.18$ per day, corresponding to infected cell life-span of 5.5 days. The viral infectivity rate $\beta =1.85\times 10^{-6}$ ml/virus per day is 296-times higher than that of HEP group, but the average viral production $p=25.3$ virion per day, is 77-times lower. The HBsAg expansion rate varies among the HEP/HIS mice, with the average $r_S=0.8 \times 10^{-4}$ HBsAg per infected cell per day, 6.7-times lower than that of the HEP mice.

For the ten mice in the HEP/HIS group for which the killing rate is non-zero, we computed the basic reproduction number

4 $$\begin{aligned} R_0=K\frac{\beta p}{c\delta }, \end{aligned}$$

which accounts for the average number of secondary cell infections in a naive hepatocyte population. The average basic reproduction number is $R_0=5.26$ ( ranging between $R_0=1.02$ and $R_0=7.74$ among the ten mice ) which is similar to the $R_0$ estimate in humans Whalley et al. (2001).

To determine the amount of liver damage due to immune mediated killing in the HEP/HIS group, we computed the total liver loss

5 $$\begin{aligned} Loss=1-\frac{T+I}{K}, \end{aligned}$$

for the HEP/HIS group and found that a peak $38-95\%$ liver loss occurs in 9 out of 12 mice (see Fig. 3A), with mouse 62 experiencing $95\%$ liver loss at day 12. The liver size does not rebound to its maximum in spite of the assumed liver proliferation. Interestingly, the amount of liver loss does not correlate with the magnitude of killing rate $\delta$, but correlates weakly with $R_0$ (correlation coefficient $r=0.79$ with p<0.006).

Since the large amount of liver killing predicted by model Eq. 1 in some of the HEP/HIS mice would lead to the animal’s death and is, therefore, not realistic, we will next investigate alternative anti-viral effects, namely non-cytolytic immune responses that lead to cure and refraction state in the previously infected hepatocyte and non-cytolytic effects that lead to reduction in either viral infection or viral production. We will adjust model Eq. 1 to account for these two assumptions.

**Fig. 5:**
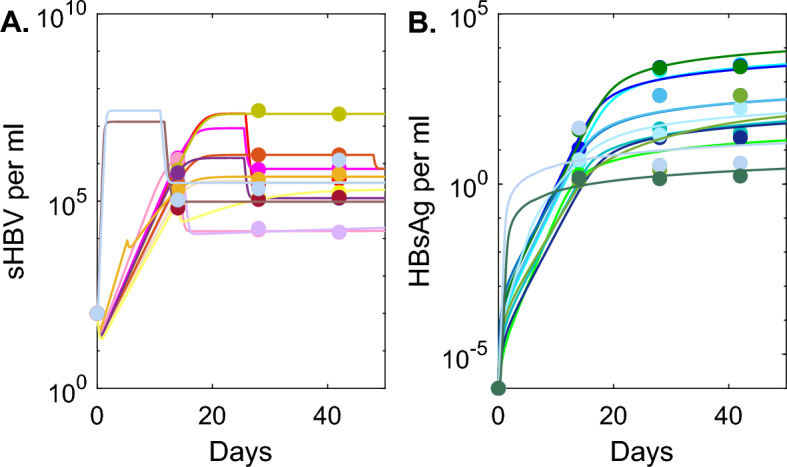
**A**: *V*(*t*) as given by model Eq. 8 versus HEP/HIS sHBV data; **B** *S*(*t*) as given by model Eq. 8 versus HEP/HIS HBsAg data. Model parameters are given in Table 5 (Color figure online)

### Refractory Cell Formation Following Cure in HEP/HIS Group

We modified model Eq. 1 by considering that cure of infected cells at rate $\rho$ results in a class of immune and refractory to reinfection liver cells, *R* Ciupe et al. (2007a). Refractory cells proliferate at rate *r* (same as the uninfected cells), and the carrying capacity for this class is *K* (same as the uninfected cells). Refractory state wanes at rate $\eta$. These extended mechanisms are shown in Fig. 1B and the interactions are modeled in the following system,

6 $$\begin{aligned} \begin{aligned} \frac{dT}{dt}&=rT(1-\frac{T+I+R}{K}) -\beta T V+\eta R,\\ \frac{dI}{dt}&= \beta T V -\delta I -\rho I,\\ \frac{dV}{dt}&= p I - cV, \\ \frac{dR}{dt}&=rR(1-\frac{T+I+R}{K}) +\rho I-\eta R,\\ \frac{dS}{dt}&=r_S I- d_S S, \end{aligned} \end{aligned}$$

with initial conditions $T(0)=K$, $I(0)=0$, $R(0)=0$, $V(0)=V_0$ and $S_0=S_0$.

In the absence of data regarding maximum liver infection, we fixed the recovery rate to $\rho =0.05$ per day, corresponding to a time in infected class before recovery of 20 days and set waning rate to $\eta =0.001$ per day Ciupe et al. (2007a). We estimated individual mouse parameters ${\textbf {p}}_{HEP/HIS}=\{\beta , r_S, \delta , p\}$ by fitting model Eq. 6 to HEP/HIS data, as before. Moreover, we estimated population level mean and standard deviation using a non-linear mixed effects modelling approach (see Fig. S2 and table S2). We found similar estimates as those of model Eq. 1 (with $\delta \ne 0$) for the infectivity rate $\beta$, the viral production rate *p* and the HBsAg production rate $r_S$. The infected cell death rate $\delta$, however, is reduced on average 3.6-times, with seven out of twelve HEP/HIS mice experiencing no liver loss, $\delta =0$ (see Table 4).

We computed the total liver loss for model Eq. 6,

7 $$\begin{aligned} Loss=1-\frac{T+I+R}{K}, \end{aligned}$$

and found no liver loss in seven mice and transient liver loss in the other five. For these mice, maximum liver loss of $11-25\%$ occurred $11-21$ days post infection before the total hepatocyte population returned to maximum values *K* (see Fig. 3B).

We found similar dynamics for the HBsAg among models Eq. 1 (with $\delta \ne 0$) and Eq. 6, with equilibrium values of $2.7-7.8\times 10^3$ copies per ml for model Eq. 1 and $2.7-6\times 10^3$ copies per ml for model Eq. 6 (see Fig. 2D versus Fig. 4B). The dynamics of sHBV, however, differ among the two models. While sHBV reaches equilibrium values $4.3\times 10^5 -2.3\times 10^7$ copies per ml that are close to the value of the virus peak for model Eq. 1 (see Fig. 2C), it drops to low equilibria $10^3-8.33\times 10^5$ copies per ml (on average one year after infection) for model Eq. 6 (see Fig. 4A).

### Antiviral Effects in the HEP/HIS Group

We modify model Eq. 1 by considering an immune-mediated antiviral response that reduces HBV production rate in the HEP/HIS group at a non-constant rate $\epsilon (t)$ in the absence of hepatocyte killing, $\delta =0$. This mechanism is shown in Fig. 1C and the interactions are modeled by the following system,

8 $$\begin{aligned} \begin{aligned} \frac{dT}{dt}&=rT(1-\frac{T+I}{K}) -\beta T V,\\ \frac{dI}{dt}&= \beta T V,\\ \frac{dV}{dt}&= (1-\epsilon (t)) p I - cV, \\ \frac{dS}{dt}&=r_S I- d_S S, \end{aligned} \end{aligned}$$

where

9 $$\begin{aligned} \epsilon (t)=\left\{ \begin{array}{cc} 0, &{} t<\tau \\ \epsilon _0, &{} t\ge \tau \end{array}\right. \end{aligned}$$

and initial conditions are $T(0)=K$, $I(0)=0$, $V(0)=V_0$ and $S_0=S_0$.

**Table 6:** AIC values for models Eq. 6 and Eq.8. Emphasized values represent the best model for that subject. Results for mice for which bold and italic values are highlighted are inconclusive

HEP/HIS group		
mouse id	AIC for Eq. 6	AIC for Eq. 8
3205	$-$2.11	$\mathbf {-10.02}$
3206	$-$4.85	$\mathbf {-9.71}$
3346	$-$3.34	$\mathbf {-9.27}$
14051	$-8.83$	$-8.33$
14062	$-$5.38	$\mathbf {-11.02}$
8061	$-$9.72	$\mathbf {-10.85}$
8044	$-$3.03	$\mathbf {-5.48}$
669	$-$8.22	$\mathbf {-10.85}$
621	$-2.20$	$-2.85$
623	$-$7.48	$\mathbf {-9.71}$
661	$-2.11$	$-2.06$
612	$-$2.78	$\mathbf {-4.09}$

We assume that parameters *r*, *c*, $d_S$ are known (see Section 3.2) and estimated individual mouse parameters ${\textbf {p}}_{HEP/HIS}=\{\beta , r_S, p, \epsilon _0, \tau \}$ by fitting model Eq. 8 to HEP/HIS data, as before. Moreover, we estimated population level mean and standard deviation using a non-linear mixed effects modelling approach (see Fig. S3 and table S3). We found an average $79\%$ reduction in viral production, occurring on average 17 days post infection (see Table 5). This leads to up to three order of magnitude reduction between peak and set points for mouse 661 and mouse 612, for which $99\%$ antiviral effect occurred 11 days post infection. For mouse 3205, for which $99\%$ reduction in viral production occurred at day 25 post infection, there was only a 36-fold decay from sHBV peak to set point (see Fig. 5A). Lastly, for mouse 14051 no reduction in the viral production is observed. The HBsAg dynamics do not change compared to the previous two models (see Fig. 5B), with average $r_S$ values similar to those in models Eq. 1 and Eq. 6.

We also considered an antiviral effect that reduces virus infectivity rate $\beta$ at non-constant rate $\sigma (t)$ in the absence of hepatocyte killing $\delta =0$. It is given by

10 $$\begin{aligned} \begin{aligned} \frac{dT}{dt}&=rT(1-\frac{T+I}{K}) - (1-\sigma (t))\beta T V+\rho I,\\ \frac{dI}{dt}&= (1-\sigma (t))\beta T V -\rho I,\\ \frac{dV}{dt}&= p I - cV, \\ \frac{dS}{dt}&=r_S I- d_S S, \end{aligned} \end{aligned}$$

where

11 $$\begin{aligned} \sigma (t)=\left\{ \begin{array}{cc} 0, &{} t<\theta \\ \sigma _0, &{} t\ge \theta . \end{array}\right. \end{aligned}$$

Model Eq. 10, however, did not fit the data well, having high residual sums of square (RSS) values for all mice. We, therefore, will not present them here.

### Model Selection

Given that under realistic biological conditions (reduced liver killing) we have two models describing different immune mechanisms for the HEP/HIS data (Eq. 6 and Eq. 8), we computed Akaike Information Criterion (AIC) values for both in order to determine which model best describes the data. We let

12 $$\begin{aligned} AIC=n\ln (\frac{1}{n}\times RSS)+2(k+1), \end{aligned}$$

where *n* is the number of data points used for data fitting and *k* is the number of parameters being estimated. For both models $n=8$ and $k=4$ for Eq. 6, $k=5$ for Eq. 8. Model selection theory says that a model with the lowest AIC best describes the data. We found that model Eq. 8 outperforms model Eq. 6 for nine mice (see Table 6 highlighted Italic). For the other three mice, however, the AIC values are similar (see Table 6 highlighted Bold). This means that we cannot uniquely select a model that best describes the data in all mice.

## Discussion

In this study, we developed within-host mathematical models of HBV infection that describe the mechanisms behind differences in viral kinetics between HEP mice (engrafted with human hepatocytes) and HEP/HIS mice (dually co-engrafted with human hepatocytes and components of a human immune system) Hogan et al. (2023). They are adaptations of previous within-host models developed for humans and chimpanzees infections Ciupe et al. (2007b, 2007a) and all have key HBV-specific components. Specifically, given the hepatocyte tropism for HBV, a term describing fast liver proliferation following liver stress and death has been included Summers et al. (2003). Moreover, given that HBV is a DNA virus that does not always integrate in the genome of an infected cell, a cure of infected cells term has been considered Guidotti et al. (1999); Wieland et al. (2004). Lastly, given that HBV does not kill infected cells by itself, only immune-mediated infected cell death was considered Thimme et al. (2003).

We fitted the models with measured sHBV and HBsAg data from seven mice in HEP group and twelve mice in HEP/HIS group reported in Hogan et al. 2023 and estimated several key parameter values for each group. Since empirical data showed reduction in both sHBV and HBsAg in mice from HEP/HIS group compared to those in the HEP group Hogan et al. (2023), we assumed that the grafted human immune system mounts an antiviral response against HBV. We determined inter group variability by assuming no antiviral responses for the HEP group (model Eq. 1 with $\delta =0$) and modeling three possible immune functions for the HEP/HIS group: cytolytic immune responses leading to cell death (model Eq. 1 with $\delta \ne 0$); non-cytolytic immune responses leading to cure and refraction to reinfection of previously infected cells (model Eq. 6); and delayed non-specified antiviral effect reducing viral production weeks after infection (model Eq. 8).

We found similar dynamics for the seven mice in the HEP group, with fast expansion of sHBV (and HBsAg) reaching high equilibria of $6.7\times 10^7- 7.4\times 10^8$ sHBV per ml ($2.7\times 10^3-1.2\times 10^4$ HBsAg per ml), $2-3$ weeks post infection. By contrast, there is large variability within the HEP/HIS group, regardless of which immune function is being modeled. All the models and data herein predict reduction in both sHBV and HBsAg levels. The magnitude of the HBsAg reduction is similar among models, reaching $2.7-7.7\times 10^3$ copies per ml equilibrium for model Eq. 1, $2.7-6\times 10^3$ copies per ml equilibrium for model Eq. 6 and $2-5\times 10^3$ copies per ml equilibrium for model Eq. 8. The magnitude of the sHBV reduction, however, depends on the model considered, reaching $4.3\times 10^5 -2.3\times 10^7$ copies per ml equilibrium for model Eq. 1, $10^3-8.33\times 10^5$ copies per ml equilibrium for model Eq. 6 and $1.6\times 10^4-2.1\times 10^7$ copies per ml equilibrium for model Eq. 8. The time to reach sHBV equilibrium is 2-3 weeks for models Eq. 1 and Eq. 8 and one year for Eq. 6.

Our goal was to select the immune response model that best describes the data in the immune competent group HEP/HIS. Model Eq. 1 (with $\delta \ne 0$) predicted high percent liver loss (up to 95%) in some mice. That would lead to mice death, which is not in agreement with experimental data showing limited evidence of liver injury Hogan et al. (2023). Moreover, Eq. 1 predicts oscillatory behavior in some of the mice, which is not seen in set-point data. Hence Eq. 1 (with $\delta \ne 0$) can be eliminated. Model Eq. 8, which predicted that antiviral effects result in reduction of up to $99\%$ viral production (one to three weeks post infection) best describe the data of nine mice (Table 6). This is reminiscent of a previous report on early acute hepatitis C virus kinetics in immuno competent chimpanzees Dahari et al. (2005). In the remaining three mice, both models Eqs. 8 and 6, which assumes non-cytolytic immune function with infected cells being cured and becoming refractory to reinfection, explain the data. Hence it is inconclusive if non-cytolytic immune responses, antiviral effects or a combination of the two is needed for reduction in the sHBV and serum HBsAg concentrations, as observed in the HEP/HIS mice.

Our study has several limitations. First, we assumed that the reduction in viral production happens instantaneously, and modeled it using a step function. A more realistic approach would be to model gradual decay based on non-hepatotoxic processes, such as interferon or lymphotoxin beta induced activation of cytidine deaminases acting on cccDNA. More data is needed to determine the shape of such a continuous antiviral effect and overall sHBV and HBsAg dynamics, which can have complex patterns of decay, as seen recently in HBV infected severe combined immunodeficient mice Hailegiorgis et al. (2023); Ishida et al. (2018). Second, we assumed that the immune processes are mutually exclusive. That is, of course, not the case and a combination of mechanisms may be responsible for the observed differences in the sHBV and HBsAg dynamics in the HEP and HEP/HIS groups. The sparcity of the data, however, prevents us from modeling them at the same time. Lastly, we made several assumptions for our fixed parameters, who can influence our results. In particular, we assumed a long HBsAg half-life of 69 days (based on preliminary fitting). Previous work showed high variability in the estimates of the half-life of serum HBsAg, ranging from a few hours up to 38 days Neumann et al. (2010); Chulanov et al. (2003); Kadelka et al. (2021); Shekhtman et al. (2018); Hershkovich et al. (2023); Shekhtman et al. (2020). Future work is needed to determine whether the longer half-life is a characteristic of our animal model.

In conclusion, we have developed several within-host models of HBV infection and used them to predict which immune mechanisms led to a reduction in sHBV and HBsAg in HEP/HIS mice compared to HEP mice. We validated the models against experimental data and found that both non-cytolytic antiviral mechanisms (yet to be identified) that lead to large reduction in viral production 1-3 weeks after the infection and/or non-cytolytic infected cell cure that lead to the emergence of cells refractory to reinfection may be responsible for improved outcomes. Further experimental and theoretical efforts are needed to dissect the human immune response mechanisms of viral control that can guide interventions.

### Supplementary Information

Below is the link to the electronic supplementary material.

Supplementary file 1 (pdf 247 KB)

## Data Availability

The code used in the manuscript and the data will be available on SMC github account uppon publication.
