# Supplementary material for: Mathematical Models of Early Hepatitis B Virus Dynamics in Humanized Mice
Source: Bull Math Biol. 2024 Apr 9;86(5):53. doi: 10.1007/s11538-024-01284-2 (PMC11003933; doi:10.1007/s11538-024-01284-2)
Supplement: Supplementary file 1 — (pdf 247 KB) [file 11538_2024_1284_MOESM1_ESM.pdf]

Mathematical models of early hepatitis B virus dynamics in humanized mice -Supplementary material

We estimate the population level mean and standard deviation for parameters $\mathbf{p} = \{\beta, r_S, \delta, p\}$ for models **Eq. 1** and **Eq. 6** and parameters $\mathbf{p} = \{\beta, r_S, p, \eta, \tau\}$ for model **Eq. 8** using a non-linear mixed effects modelling approach that utilizes Stochastic Approximation Estimation-Maximization (SAEM) algorithm in Monolix [1]. The results are given in Fig. S1 and table S1 for model **Eq. 1**, in Fig. S2 and table S2 for model **Eq. 6** and in Fig. S3 and table S1 for model **Eq. 8**.

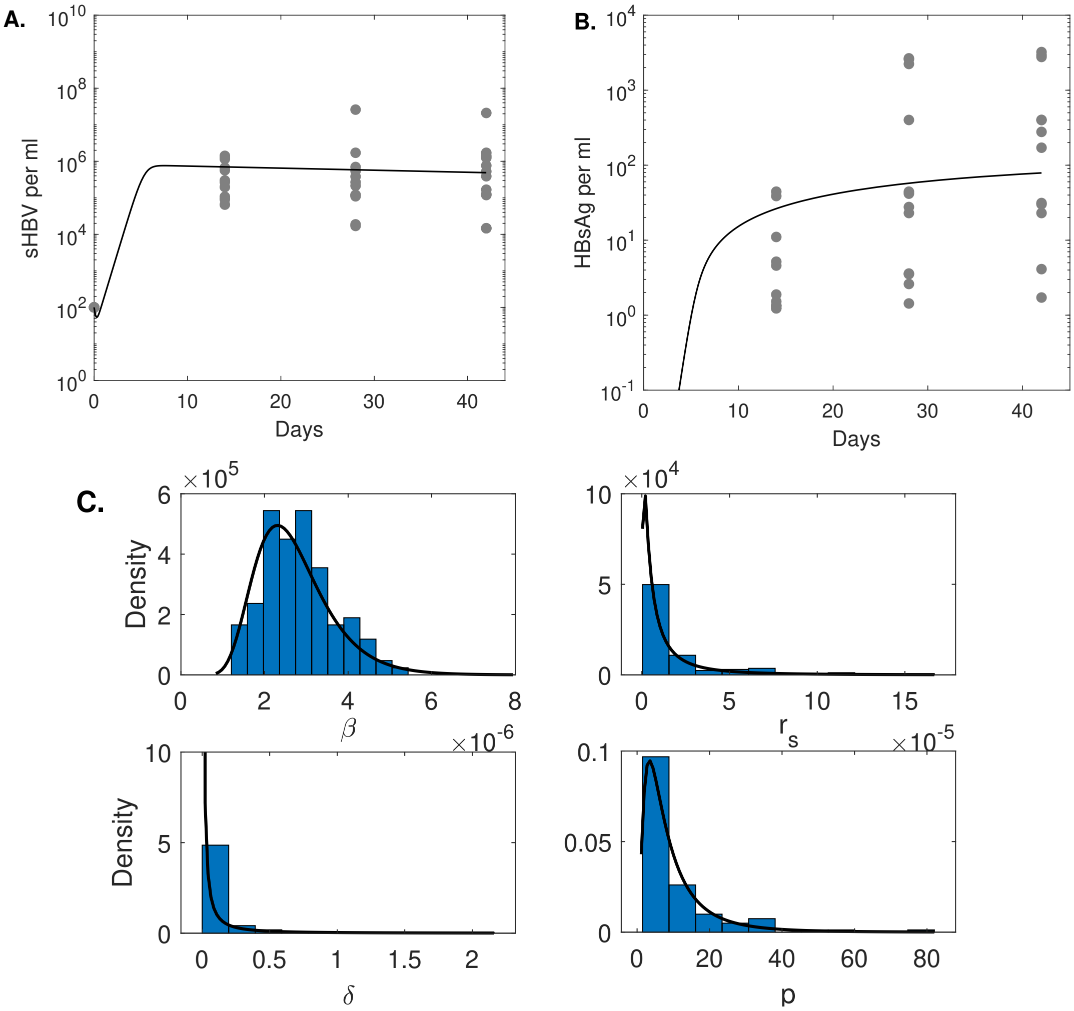

Figure S1: **A:** sHBV population fit given by model **Eq. 1** (solid line) versus data (circles); **B:** HBsAg population fit given by model **Eq. 1** (solid line) versus data (circles); **C:** Parameter distributions from fitting model **Eq. 1** to population data.

**Parameter**	**Mean**	**STDV**
β	2.57×10^{-6}	0.33
r_S	6.68×10^{-6}	1.37
δ	0.012	2.51
p	7.1	0.85
**AIC**	222.3	

Table S1: Mean and stdv for parameters β, r_S, δ, p found by fitting model **Eq. 1** to data using the SAEM algorithm in Monolix.

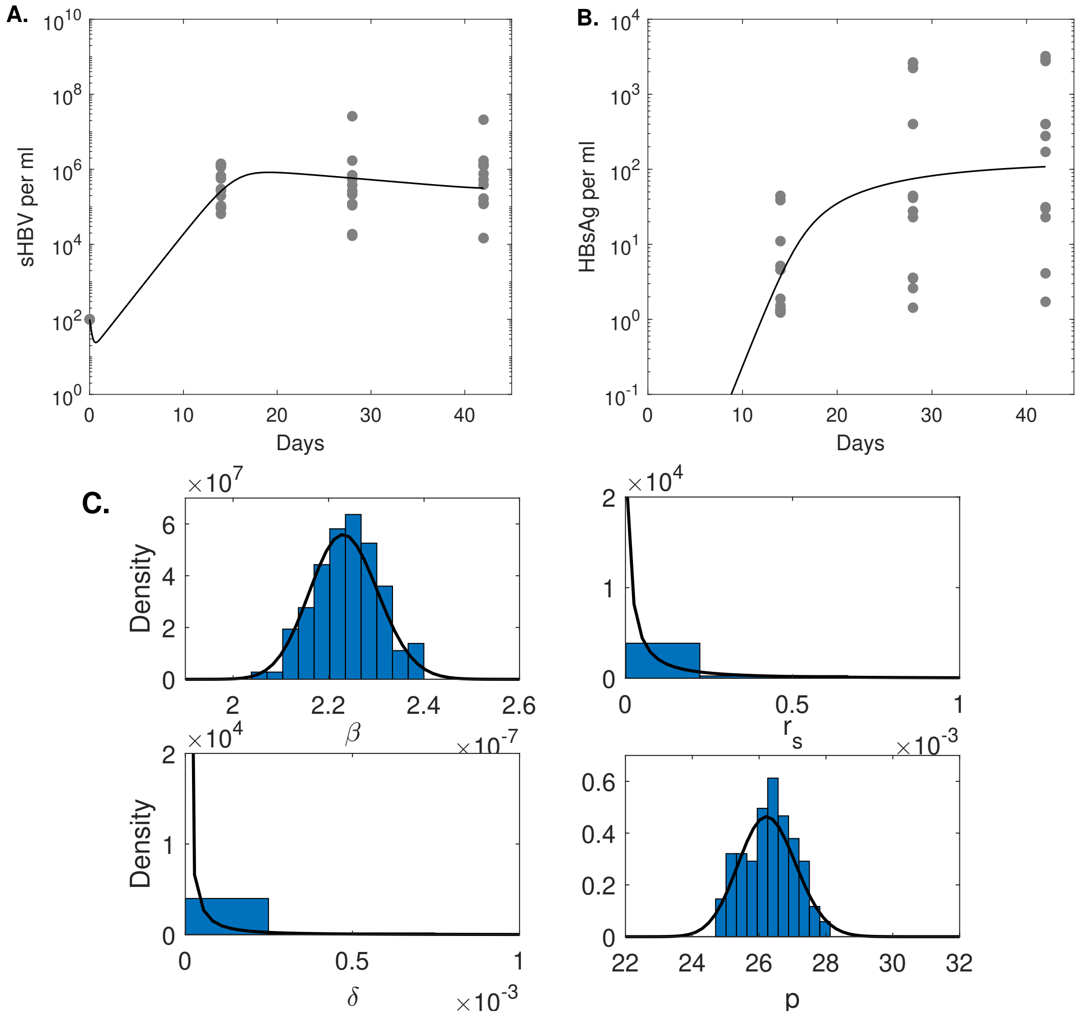

Figure S2: **A:** sHBV population fit given by model **Eq. 6** (solid line) versus data (circles); **B:** HBsAg population fit given by model **Eq. 6** (solid line) versus data (circles); **C:** Parameter distributions from fitting model **Eq. 6** to population data.

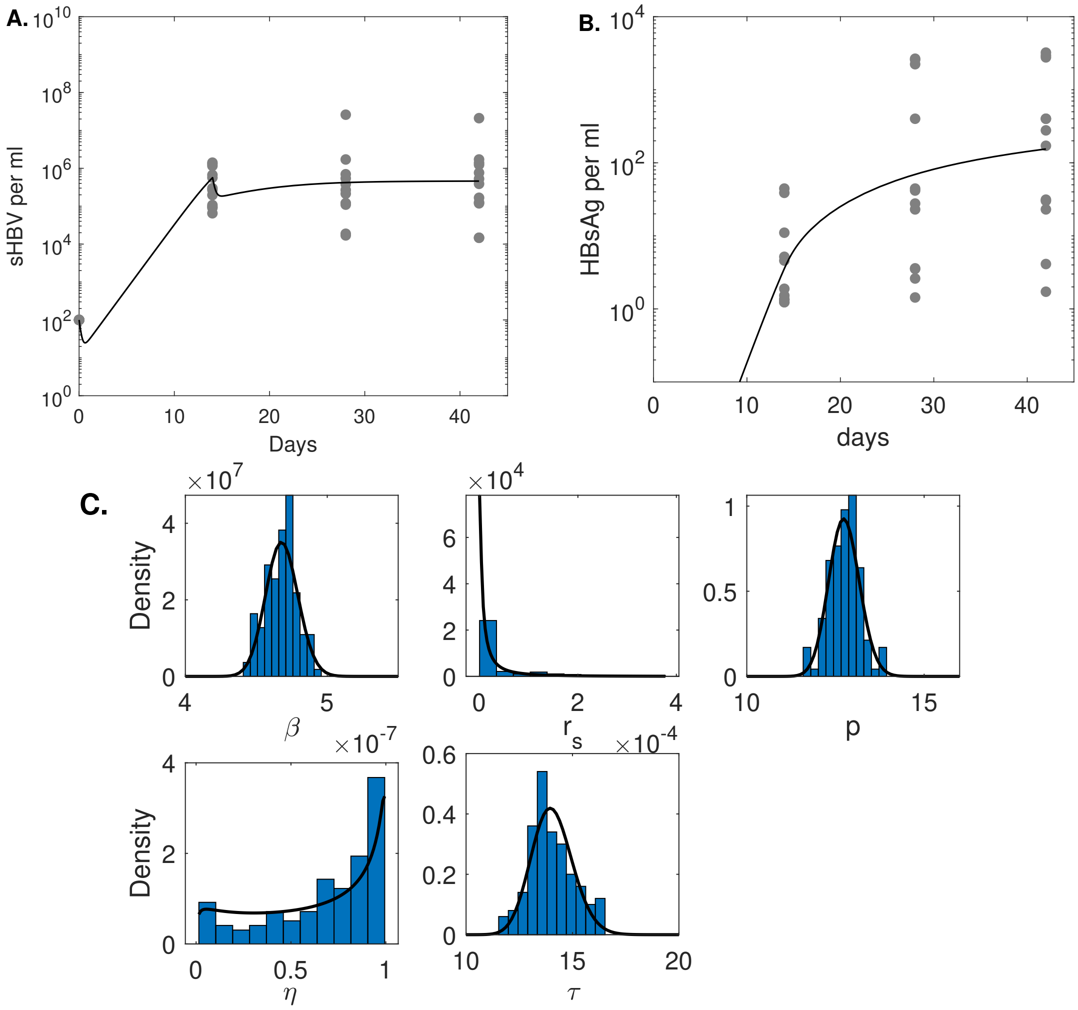

Figure S3: **A:** sHBV population fit given by model **Eq. 8** (solid line) versus data (circles); **B:** HBsAg population fit given by model **Eq. 8** (solid line) versus data (circles); **C:** Parameter distributions from fitting model **Eq. 8** to population data.

**Parameter**	**Mean**	**STDV**
β	2.23×10^{-7}	0.031
r_S	4.47×10^{-5}	1.79
δ	1.15×10^{-5}	2
p	26.2	0.032
**AIC**	224	

Table S2: Mean and stdv for parameters β, r_S, δ, p found by fitting model **Eq. 6** to data using the SAEM algorithm in Monolix.

Parameter	Mean	STDV
β	4.68×10^{-7}	0.024
r_S	1.1×10^{-5}	1.65
p	12.7	0.033
η	0.76	2
τ	14	0.07
**AIC**	193	

Table S1: Mean and stdv for parameters β, r_S, p, η, τ found by fitting model **Eq. 8** to data using the SAEM algorithm in Monolix.

References

[1] Monolix version 2019r2. Antony, France: Lixoft SAS, 2019.
